# Effect of previous wedge resection for interstitial pregnancy on pregnancy and neonatal outcomes following frozen-thawed embryo transfer (FET) cycles of IVF/ICSI: a retrospective study

**DOI:** 10.1186/s12958-022-00896-4

**Published:** 2022-02-01

**Authors:** Shengluan Tang, Tong Du, Jialyu Huang, Hongjuan Ye, Ming Zhao, Jiaying Lin, Yanping Kuang

**Affiliations:** 1 Department of Assisted Reproduction, Shanghai Ninth People’s Hospital, Shanghai Jiao Tong University School of Medicine, 639 Zhizaoju Rd, Shanghai, 200011 China; 2 Department of Reproductive Medicine Center, Shanghai East Hospital, Tongji University School of Medicine, Shanghai, China; 3 Shanghai Towako Hospital, No. 477, Fute West 1st Road, Shanghai, China

**Keywords:** Interstitial pregnancy, Wedge resection, Frozen embryo transfer, Pregnancy outcomes, Neonatal outcomes

## Abstract

**Objective:**

The present study aimed to evaluate pregnancy and neonatal outcomes in women, with a previous history of wedge resection for interstitial pregnancy, in frozen-thawed embryo transfer (FET) cycles of IVF/ICSI.

**Methods:**

The present study involved a retrospective case-control assessment of 75 cases and 375 control subjects over 6 years in a single center. To compare pregnancy and neonatal outcomes between cases, treated using wedge resection, and controls without any previous history of ectopic pregnancy, propensity score matching (1:5) was utilized. The study also compared subgroups in the case group.

**Results:**

Women with previous wedge resection exhibited higher rates of ectopic pregnancy and uterine rupture rate as compared to control subjects (9.1% vs 1.3%, *P* = 0.025 and 4.5% vs 0%, *P* = 0.035, respectively). No statistically significant differences were recorded between the two cohorts with regard to clinical pregnancy rate, live birth rate, and neonatal outcomes. For pregnancy type subgroup analysis, Z-score and rates of large for gestational age were recorded to be significantly lower in twin pregnancy subgroup when compared with singleton pregnancy subgroup (0.10 (− 0.59, 0.25) vs 0.50 (− 0.97, 1.39), *P* = 0.005; 4.5% vs 26.1%, *P* = 0.047, respectively).

**Conclusion:**

The results of the present study indicated that previous wedge resection correlated to a higher risk of ectopic pregnancy and uterine rupture. However, it might not be related to an increased risk of adverse neonatal outcomes. The study recommended cesarean section in these patients. Further studies are required to verify the validity of current recommendations.

**Supplementary Information:**

The online version contains supplementary material available at 10.1186/s12958-022-00896-4.

## Introduction

Interstitial pregnancy represents a rare subtype of ectopic pregnancies, wherein fertilized ovum gets implanted in the proximal portion of the fallopian tube that traverses the myometrium. It accounts for 2–4% of all ectopic pregnancies. Importantly, the mortality rate associated with interstitial pregnancy is seven times greater than that of ectopic pregnancies [1, 2]. A series of treatment modalities have been reported in previous studies. Traditionally, interstitial pregnancies were treated with hysterectomy or wedge resection, wherein the gestational sac was removed along with neighboring uterine myometrium [3–6].

Following wedge resection, uterine rupture is a major concern in such patients, primarily owing to the appearance of the uterine scar after the surgery [7–9], which might lead to early uterine rupture. However, the risk of uterine rupture has not been extensively studied. Furthermore, it has also been reported that wedge resection might affect future fertility [10]. However, only very few studies assessed reproductive outcomes in women subjected to wedge resection for interstitial pregnancy [11, 12]. In fact, there are no reports on neonatal outcomes in such cases. Importantly, no studies are available on subsequent pregnancy and neonatal outcomes in women treated with assisted reproductive technology (ART) after wedge resection. Consequently, not much information is available to assist the management of patients treated using ART. It has been previously reported that a part of the patients subjected to wedge resection could not conceive naturally, and the tubal function of natural conception was found to be different from that of ART. Therefore, it is necessary/important to study pregnancy outcomes and neonatal outcomes in the patients treated using ART. The center where the present study was conducted usually follows a freeze-all strategy, as the risk of ovarian hyperstimulation syndrome and ectopic pregnancy after frozen-thawed embryo transfer (FET) have been reported to be lower as compared to fresh embryo transfers [13–17]. Therefore, the present study aimed to evaluate the effect of previous wedge resection, conducted/performed for interstitial pregnancy, on pregnancy and neonatal outcomes in women undergoing FET. In particular, a case-control study was conducted.

## Materials and methods

### Study subjects

A retrospective cohort study was conducted from February 2014 to May 2019 at the Department of Assisted Reproduction of Shanghai Ninth People’s Hospital. Patients who were previously treated by wedge resection for interstitial pregnancy (*n* = 75) and control subjects (*n* = 19,423) without previous ectopic pregnancy were identified from their electronic medical records in the FET cycles (the flowchart is shown in Supplemental Figure 1). As the surgical history was obtained through medical history inquiry, the wedge resections were performed by different surgeons in different hospitals. The following patients were excluded: 1) those with uterine abnormities (e.g., unicornuate uterus and bicornuate uterus) or other types of uterine surgery (e.g., myomectomy and cesarean section); 2) those with a previous diagnosis of diabetes, hypertension, or thyroid disorders; 3) those for whom the cycle records with core data were missing.

### Study design

To evaluate the impact of previous wedge resection in pregnancy and neonatal outcomes, propensity score matching (matched ratio 1:5) was performed on the wedge resection group (WR group, women with previous wedge resection) and the non-ectopic pregnancy group (NonEP group, women without previous ectopic pregnancy).

First, the pregnancy outcomes were compared between the WR and NonEP groups in the initial FET cycles after matching the propensity scores. Variables selected for analysis included the clinical pregnancy rate, biochemical pregnancy rate, ectopic pregnancy rate, miscarriage rate, implantation rate, multiple gestation rate, live birth rate, multiple birth rate, uterine rupture rate, mode of delivery, and gestational age at delivery.

Second, the neonatal outcomes were compared between the two groups among singleton infants born from the first clinical pregnancy cycles so as to eliminate the effect of repeated cycles on the neonatal outcomes. Z-score, low birth weight (LBW), high birth weight (HBW), small for gestational age (SGA), large for gestational age (LGA), congenital malformations, and early neonatal death were selected as parameters for analyses.

In addition, we performed a subgroup analysis of the WR group. The WR group was classified into a singleton pregnancy subgroup and a twin pregnancy subgroup. The pregnancy outcomes and the neonatal outcomes were compared between the subgroups in the first clinical pregnancy cycle.

### Treatment

Procedures of IVF/ICSI, embryo culture, endometrial preparation, and embryonic transfer have been described in our previous publications [18, 19]. In short, the Cummins’ criteria were used to grade day 3 embryos [20]. Grade I and II embryos that were deemed to be high-quality were qualified for vitrification. Embryos that were classified as low grade (Grade III and IV) underwent extended culturing and evaluation until Day 7. Blastocysts during this stage were graded according to the Gardner and Schoolcraft system [21]. Blastocysts were graded as 3 BC or better to be frozen on days 5–6, and only Grade 3CC or better embryos were frozen on day 7. The endometrial preparation protocol included natural cycles, hormone replacement therapy, and stimulated cycles. For patients with regular menstrual cycles, natural cycles were applied; hormone replacement therapy was applied in patients with thin endometria during other FET cycles; in patients with irregular menstrual cycles, the stimulated cycles were used.

### Outcomes

Variables assessed for reproductive outcomes were defined based on the ART terminology [22]. Ectopic pregnancy (also included in clinical pregnancy) was defined as a pregnancy in which implantation takes place outside the uterine cavity. The implantation rate was defined as the number of gestational sacs observed divided by the number of embryos transferred. The live birth delivery rate was defined as the number of deliveries with at least one live-born infant per 100 embryo transfer cycles. The Z-score was selected to calculate the birth weight modified for gestational age and gender, as follows: Z-score = (x - μ)/σ (x represents the birth weight, μ represents the mean baby weight for equal gestational age and gender, and σ represents the standard deviation of the equal gestational age and gender). LBW and HBW were determined as birth weights < 2500 g and > 4500 g, respectively. SGA and LGA were determined as birth weights <10th percentiles and > 90th percentiles, respectively. The Z-scores and birth weight percentiles were dependent on birth weight reference percentiles for Chinese singleton and twin newborns [23, 24]. Congenital malformations were based on the International Classification of Diseases Q codes [25].

### Statistical analysis

Propensity score matching was performed on the WR and NonEP groups through the nearest neighbor matching within 0.15 caliper width (matched ratio 1:5). Propensity scores were assessed by using a logistic regression model on the baseline characteristics [26], including the age, body mass index (BMI), gravidity, parity, year of treatment (2014–2015, 2016–2017, and 2018–2019), infertility duration, infertility causes, endometrial preparation protocol, endometrial thickness, the number of embryos transferred, and the stage of embryo (cleavage stage and blastocyst).

All statistical analysis was performed with the SPSS software v.26.0. For continuous variables, the normality was tested by the Shapiro-Wilk test and Q-Q plots. Continuous variables that were normally distributed were described by mean with standard deviation, otherwise, they were described by median (four Quartiles). Since normality (and homogeneity of variance) assumptions were not satisfied in all continuous variables, they were described by median (four Quartiles) and the equivalent non-parametric test was applied for comparison. Categorical variables were represented in several cases with percentages and compared via chi-squared tests or Fisher’s exact test, as deemed appropriate. *P* < 0.05 was considered to indicate statistical significance. Logistic regression models were utilized to calculate the adjusted odds ratios (ORs) and 95% confidence intervals (CIs)..

## Results

### Pregnancy outcomes

**Fig. 1:**
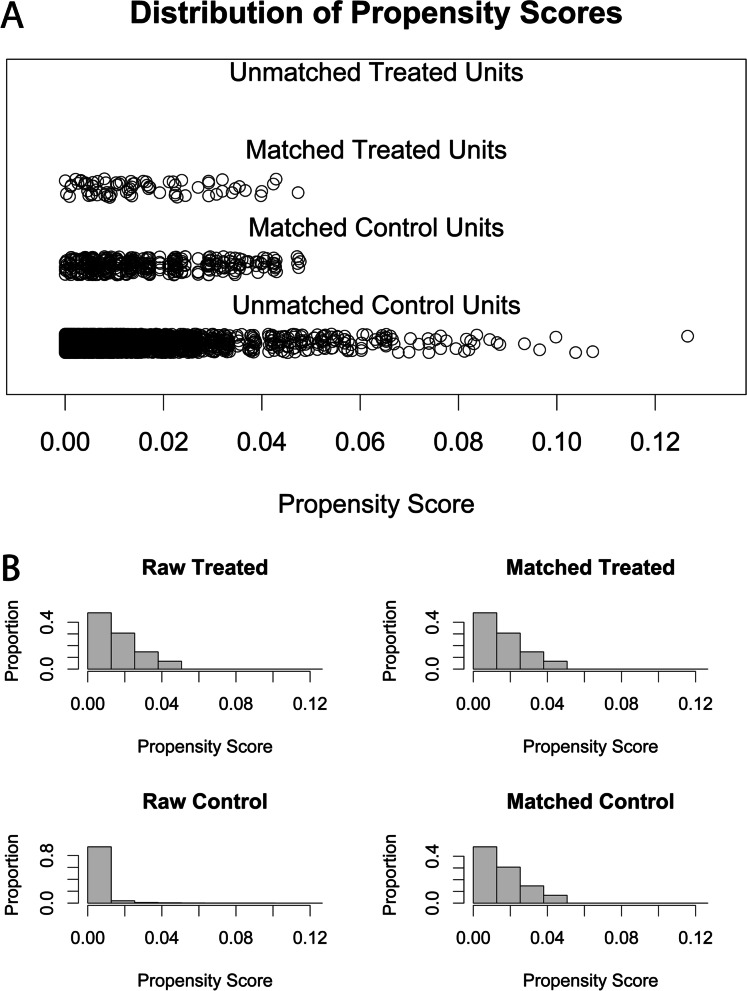
Propensity score matching for WR group and NonEP group. The distributions of the propensity score (**A**) and histogram of propensity scores (**B**) indicated a balance between the compared cohorts

The present study involved the recruitment of cases and controls, retrieved from a cohort of 24,374 women who were subjected to IVF/ICSI and FET at Shanghai Ninth People’s Hospital, from February 2014 to May 2019. The study involved a total of 75 women with the previous history of wedge resection. For each case, five control subjects were selected as a reference by propensity score matching, which resulted in a study population of 450 women. The distributions and histograms for propensity scores, before and after propensity score matching, are shown in Fig. 1. As shown in Fig. 1, a balance existed between the compared cohorts. For these 75 patients, 46 patients (61.3%) had surgical locations towards the right side, while 29 patients (38.7%) had surgical locations towards the left side. Importantly, laparoscopy was performed in 46 cases (61.3%), while laparotomy was conducted in 29 cases (38.7%). Interestingly, methotrexate treatment was used in three of these patients, two of these were subjected to laparoscopy and one underwent laparotomy.

The baseline characteristics for the subjects are listed in Table 1, while pregnancy outcomes between matched WR vs NonEP groups are displayed in Table 2. WR group exhibited a significantly higher rate of ectopic pregnancy as compared to the NonEP group (9.1% vs 1.3%, *P* = 0.025). In particular, four recurrent ectopic pregnancies were recorded in the WR group. Among these, one case each of contralateral tubal ectopic pregnancy, contralateral interstitial pregnancy, ipsilateral interstitial pregnancy, and heterotopic pregnancy (contralateral interstitial that ended up with miscarriage) were recorded. Besides this, uterine rupture rate was also recorded to be significantly higher in the WR group as compared to the NonEP group (4.5% vs 0%, *P* = 0.035). No statistically significant differences were recorded between the two cohorts in terms of clinical pregnancy rate, biochemical pregnancy rate, miscarriage rate, implantation rate, multiple gestation rate, live birth rate, multiple birth rate, mode of delivery, and gestational age at the time of delivery. The cesarean section rate was recorded to be 83.3% (30/36) and 76.3% (116/152) in the WR group and NonEP group, respectively. Since subsequent delivery of the patients was not performed in the hospital where this study was conducted, the information regarding the indications for the mode of delivery in the WR group is unavailable. Importantly, two cases (both singleton pregnancies) presented with uterine rupture in the WR group. In particular, the first case underwent left laparoscopic wedge resection for interstitial pregnancy 5 years ago. This patient was subjected to acute cesarean section due to intra-abdominal bleeding at 29 weeks of gestation, and the site of uterine rupture was located in the scar of earlier wedge resection. The baby boy died of neonatal asphyxia days after birth. In comparison to this, the second case received right laparoscopic wedge resection 4 years ago, and uterine rupture was observed during elective cesarean section at 37 weeks, which resulted in the delivery of a healthy baby boy.

**Table 1:** Baseline characteristics of the matched groups and the pregnancy type subgroup

Characteristics	WR group	NonEP group	*P*	Singleton subgroup	Twin subgroup	*P*
n	75	375		50	11	
Age (years)	36(33,40)	36(32,40)	0.467	36(33,39)	36(33,37)	0.785
BMI (kg/m2)	22.99(20.20,25.40)	22.43(20.43,24.52)	0.282	23.06(20.41,25.45)	21.70(19.95,23.53)	0.404
Year of treatment (%)			0.914			0.700
2014–2015	28.0(21/75)	29.9(112/375)		28.0(14/50)	18.2(2/11)	
2016–2017	34.7(26/75)	35.2(132/375)		34.0(17/50)	27.3(3/11)	
2018–2019	37.3(28/75)	34.9(131/375)		38.0(19/50)	54.5(6/11)	
Infertility duration (years)	2(1,3)	2(0,3)	0.567	2(0,3)	2(0,4)	1.000
Gravidity			0.879			1.000
0	8.0(6/75)	8.5(32/375)		10.0(5/50)	9.1(1/11)	
≥ 1	92.0(69/75)	91.5(343/375)		90.0(45/50)	90.9(10/11)	
Parity			0.592			1.000
0	78.7(59/75)	81.3(305/375)		80.0(40/50)	81.8(9/11)	
≥ 1	21.3(16/75)	18.7(70/375)		20.0(10/50)	18.2(2/11)	
Tubal infertility (%)	98.7(74/75)	98.1(368/375)	1.000	98.0(49/50)	100(11/11)	1.000
PCOS (%)	6.7(5/75)	5.9(22/375)	0.790	6.0(3/50)	18.2(2/11)	0.218
Endometriosis (%)	5.3(4/75)	6.4(24/375)	1.000	4.0(2/50)	18.2(2/11)	0.146
Male factor infertility (%)	10.7(8/75)	10.9(41/375)	0.946	12.0(6/50)	9.1(1/11)	1.000
Endometrial preparation (%)			0.625			0.629
Natural cycles	24.0(18/75)	29.1(109/375)		22.0(11/50)	9.1(1/11)	
HRT cycles	37.3(28/75)	36.8(138/375)		36.0(18/50)	36.4(4/11)	
Stimulated cycles	38.7(29/75)	34.1(128/375)		42.0(21/50)	54.5(6/11)	
Endometrial thickness (mm)			0.752			0.233
< 8	9.3(7/75)	7.2(27/375)		8.0(4/50)	0(0/0)	
8–11	61.3(46/75)	65.1(244/375)		66.0(33/50)	45.5(5/11)	
> 11	29.3(22/75)	27.7(104/375)		26.0(13/50)	54.5(6/11)	
No. of embryos transferred			0.286			**0.018**
1	48.0(36/75)	41.3(155/375)		60.0(30/50)	18.2(2/11)	
2	52.0(39/75)	58.7(220/375)		40.0(20/50)	81.8(9/11)	
Stage of embryo			0.190			0.224
Cleavage stage	82.5(94/114)	87.1(518/595)		65.7(46/70)	80.0(16/20)	
Blastocyst	17.5(20/114)	12.9(77/595)		34.3(24/70)	20.0(4/20)	

*WR group* patients with a history of wedge resection; *NonEP group* patients without a history of ectopic pregnancy; *PCOS* polycystic ovarian syndrome. Non-normal distribution quantitative data are presented as median (four Quantile). Qualitative data are presented as % (n/N). *P* < 0.05 was considered statistically significant

**Table 2:** The pregnancy and neonatal outcomes of the matched groups

Pregnancy outcomes (%)	WR group	NonEP group	*P*
Clinical pregnancy	58.7(44/75)	50.4(189/375)	0.191
OR (95% CI)	1.53(0.90–2.61)	Reference	0.118
Biochemical pregnancy	2.7(2/75)	4.8(18/375)	0.550
OR (95% CI)	0.49(0.10–2.29)	Reference	0.361
Ectopic pregnancy	9.1(4/44)	1.3(3/189)	**0.025**
OR (95% CI)	12.60(0.93–170.78)	Reference	0.057
Miscarriage	11.4(5/44)	19.0(36/189)	0.228
OR (95% CI)	0.59(0.20–1.71)	Reference	0.326
Implantation	43.9(50/114)	38.7(230/595)	0.298
OR (95% CI)	1.29(0.76–2.18)	Reference	0.348
Multiple gestation	18.2(8/44)	22.2(42/189)	0.557
OR (95% CI)	0.89(0.33–2.43)	Reference	0.822
Live birth	48.0(36/75)	40.5(152/375)	0.231
OR (95% CI)	1.47(0.87–2.50)	Reference	0.154
Multiple birth	18.2(8/44)	19.6(37/189)	0.833
OR (95% CI)	1.08(0.39–2.98)	Reference	0.883
Uterine rupture	4.5(2/44)	0(0/0)	**0.035**
OR (95% CI)	–	–	
Mode of delivery			0.363
Vaginal	16.7(6/36)	23.7(36/152)	
Cesarean section	83.3(30/36)	76.3(116/152)	
Gestational age at delivery			0.289
< 28	12.2(5/41)	19.1(36/188)	
28 < age < 37	19.5(8/41)	11.7(22/188)	
37 < age < 42	68.3(28/41)	69.1(130/188)	
Neonatal outcomes (%)	WR group	NonEP group	P
Live born infants (n)	48	178	
Z-score	0.55(−0.06,1.52)	0.29(−0.32,0.88)	0.084
Low birth weight	6.3(3/48)	5.1(9/178)	0.721
High birth weight	0(0/0)	0.6(1/178)	1.000
Small for gestational age	2.1(1/48)	6.2(11/178)	0.469
Large for gestational age	27.1(13/48)	15.7(28/178)	0.070
Congenital malformations	0(0/0)	1.1(2/178)	1.000
Early neonatal death	2.1(1/48)	0(0/178)	0.212

*WR group* patients with a history of wedge resection; *NonEP group* patients without a history of ectopic pregnancy; *OR* adjusted odds ratio. One heterotopic pregnancy in the WR group ended up with miscarriage. Two heterotopic pregnancies were observed in the NonEP group, resulting in one miscarriage and one full-term baby. Statistically significant results are marked in bold (*p* < 0.05)

Assessment of pregnancy type for subgroup analysis divided WR group into singleton pregnancy subgroup and twin pregnancy subgroup, which comprised of 50 and 11 women, respectively. The baseline characteristics of the two subgroups are displayed in Table 1. Most baseline patient characteristics were found to be similar between the two subgroups, with exception of the number of embryos transferred. In particular, the number of embryos transferred in the case of the twin subgroup was higher as compared to that in the singleton subgroup (*P* = 0.018). Pregnancy outcomes are shown in Table 3, which included miscarriage rate, preterm birth rate, full-term birth rate, post-term birth rate, uterine rupture rate, and mode of delivery. The preterm birth rate was found to be significantly higher in the twin pregnancy subgroup than in the singleton pregnancy subgroup (54.5% vs 8.0%, *P* = 0.001).

**Table 3:** The pregnancy and neonatal outcomes of the pregnancy type subgroups

Pregnancy outcomes (%)	Singleton subgroup	Twin subgroup	P
n	50	11	
Miscarriage	8.0(4/50)	0(0/11)	1.000
Preterm birth	8.0(4/50)	54.5(6/11)	**0.001**
Full-term birth	84.0(42/50)	45.5(5/11)	**0.013**
Post-term birth	0(0/50)	0(0/11)	–
Uterine rupture	4.0(2/50)	0(0/11)	1.000
Mode of delivery			0.182
Vaginal	19.6(9/46)	0(0/11)	
Cesarean section	80.4(37/46)	100(11/1)	
Neonatal outcomes (%)	Singleton subgroup	Twin subgroup	P
Live born infants (n)	46	22	
Z-score	0.50(−0.97,1.39)	0.10(−0.59,0.25)	**0.005**
Low birth weight	6.5(3/46)	63.6(14/22)	**< 0.001**
High birth weight	0(0/46)	0(0/22)	–
Small for gestational age	2.2(1/46)	9.1(2/22)	0.243
Large for gestational age	26.1(12/46)	4.5(1/22)	**0.047**
Congenital malformations	0(0/46)	9.1(2/22)	0.101
Early neonatal death	2.2(1/46)	0(0/22)	1.000

Comparisons were made using chi-square test or Fisher’s exact test as appropriate. Statistically significant results are marked in bold (*p* < 0.05)

### Neonatal outcomes

To compare the WR group vs. NonEP group, 226 live-born infants were enrolled, which involved 48 and 178 babies in WR and NonEP group, respectively. Neonatal outcomes are shown in Table IV. No statistically significant differences were recorded during the analysis of neonatal outcomes, which included Z-score, LBW, HBW, SGA, LGA, congenital malformations, and early neonatal death. Two congenital malformations (one case each of talipes equinovarus and accessory finger) were recorded in the WR group.

Singleton pregnancy subgroup and twin pregnancy subgroup included 46 and 22 babies, respectively. Neonatal outcomes are displayed in Table IV. Z-score and rate of LGA were found to be significantly lower in twin pregnancy subgroup, when compared with singleton pregnancy subgroup (0.10 (− 0.59, 0.25) vs 0.50 (− 0.97, 1.39), *P* = 0.005; 4.5% vs 26.1%, *P* = 0.047, respectively). No statistically significant differences were found between the two subgroups in terms of SAG rate, congenital malformations, and early neonatal death.

## Discussion

The present study is first to assess the effects of previous wedge resection, performed after interstitial pregnancy, on pregnancy and neonatal outcomes following ART. The results of this study indicated that the rate of ectopic pregnancy and uterine rupture were higher in the WR group as compared to the NonEP group. Further, the results for subgroup analysis suggested that, compared with a singleton pregnancy, twin pregnancies after wedge resection might not increase the risk of uterine rupture.

No previous study is available regarding IVF outcomes in women with previous wedge resection. In fact, there is no publication for neonatal outcomes after wedge resection for interstitial pregnancy. In a previous study, Hoyos et al. [12] assessed reproductive outcomes in women with previous wedge resection for interstitial ectopic pregnancy (WRIEP), wherein pregnancy outcomes were compared in 19 patients with a history of WRIEP and pregnant-matched controls (1:3). The study showed that complication rates, delivery mode, and gestational age were comparable between the groups. In particular, four cases of recurrent ectopic pregnancy were reported (the specific situation was not described), while no incidence of uterine rupture was recorded. Svenningsen et al. [11] conducted a single-center historic cohort study that focused on fertility outcomes after wedge resection for interstitial pregnancies. The study included 26 women who underwent wedge resection and a matched reference group of 52 women (ratio 1:2). No differences were recorded between the groups in terms of subsequent pregnancy rates beyond gestational week 24. In fact, no recurrent ectopic pregnancy or uterine rupture was reported. However, the inclusion of a low patient number in these two studies limited the interpretation of the results. All other studies that explored pregnancy outcomes after wedge resection were descriptive case series [4, 10, 27–31].

Recurrent ectopic pregnancy and uterine rupture are two major concerns of pregnancy after wedge resection. In view of the low incidence of interstitial pregnancy, data available regarding recurrent ectopic pregnancy and uterine rupture after wedge resection are quite limiting. However, few studies indicated a risk of recurrent ectopic pregnancy and uterine rupture for subsequent pregnancies [3, 7–9, 32, 33], which was consistent with the results of the present study. In the present study, four recurrent ectopic pregnancies were recorded in the WR group, which suggested a higher risk of ectopic pregnancies in the following pregnancies, as compared to the NonEP group. In a previous study, Bennetot et al. reported a 2-year cumulative recurrence rate of 18.5% after salpingostomy or salpingectomy for tubal ectopic pregnancy, which is not contrary to the findings of the present study [34]. According to Egger et al., recurrent interstitial pregnancy is likely to be associated with anatomy-related risk factors, such as prior ectopic pregnancies and salpingectomy [35]. Therefore, anatomical changes arising due to wedge resection might be associated with an increased risk of recurrent ectopic pregnancy. However, data available regarding ectopic pregnancy might not be enough, and thus further research is required. Wedge resection might contribute to a uterine injury/scar, which might further increase the risk of uterine rupture as a uterine injury/scar is considered to be the most common cause of uterine rupture [33]. Therefore, the present study recommended a cesarean section for patients with previous wedge resection to decrease the risk of uterine rupture, in the absence of any contraindications.

Besides this, the present study also revealed that twin pregnancies after wedge resection might not be associated with an increased risk of uterine rupture as compared to singleton pregnancies after wedge resection. Importantly, all patients in the twin group had a cesarean section, and this might avoid rupture. The available data are still insufficient, and thus further research is required to identify optimal management strategies for twin pregnancies.

### Strengths and limitations

The present study was associated with several key strengths. The major strength was that the present study included the largest study sample size and matched control group on this particular topic so far, which provided a precious opportunity to analyze neonatal outcomes after wedge resection. No prior study is available on neonatal outcomes after wedge resection. The present study is the first to evaluate the effects of wedge resection after interstitial pregnancy on pregnancy and neonatal outcomes in patients treated with ART. Besides, propensity score matching was utilized to manage latent confounders between the WR group and NonEP group, which made outcomes conditionally independent of treatment allocation.

Since the present study involved a retrospective analysis that was conducted at a single-center, it was associated with several non-negligible limitations. Since surgical history was obtained through medical history inquiry, wedge resections were performed by different surgeons at different hospitals, which resulted in a few confounding factors. In general practice, the center involved in the present study routinely performed follow-up till 6 weeks after FET for pregnant patients, following which the patients would choose the hospital for subsequent delivery. The center would continue to follow up on the pregnancy outcome by telephonic interviews, which might lead to the unavailability of information regarding obstetrical management, neonatal management, indications for the mode of delivery, and others. In fact, almost all newborn data were obtained from question sheets, which might affect the discovery of minor birth defects. In addition to this, possible errors in data entry and patients who were lost to follow-up also acted as the limitations of the study. The number of patients with wedge resection history is still limited, primarily owing to the rarity of the condition. This might further underpower the differences in severely low rates, such as the rate of SGA. In the present case, the ability to draw definite conclusions was also restricted in subgroup analysis. In the future, well-designed randomized controlled trials are required to overcome these limitations.

## Conclusions

Altogether, the results of the present study suggested that wedge resection correlated with an increased risk of recurrent ectopic pregnancy and uterine rupture in the case of women undergoing FET. Importantly, wedge resection might not be linked to an increased risk of adverse neonatal outcomes. Thus, postoperative outcome counseling holds potential value. The present study recommended cesarean section for patients with previous wedge resection to decrease the risk of uterine rupture, in absence of any contraindications. However, further studies are required to verify the validity of these recommendations.

## Supplementary Information

**Additional file 1: Supplemental Figure S1.** Flow chart of the study.

## Data Availability

If the request is sensible, the data analyzed in the study are available from the corresponding author.

## Abbreviations

ART: Assisted reproductive technology
FET: Frozen-thawed embryo transfer
WR: Wedge resection
NonEP: Non-ectopic pregnancy
IVF: In vitro fertilization
ICSI: Intracytoplasmic sperm injection
LBW: Low birth weight
HBW: High birth weight
SGA: Small for gestational age
LGA: Large for gestational age
BMI: Body mass index
OR: Odds ratio
CI: Confidence interval
PTD: Preterm delivery
VPTD: Very preterm delivery
VLBW: Very low birthweight
PCOS: Polycystic ovary syndrome
WRIEP: Wedge resection for interstitial ectopic pregnancy
