# Supplementary figures and images for: Effect of previous wedge resection for interstitial pregnancy on pregnancy and neonatal outcomes following frozen-thawed embryo transfer (FET) cycles of IVF/ICSI: a retrospective study
Source: Reprod Biol Endocrinol. 2022 Feb 1;20:23. doi: 10.1186/s12958-022-00896-4 (PMC8805226; doi:10.1186/s12958-022-00896-4)

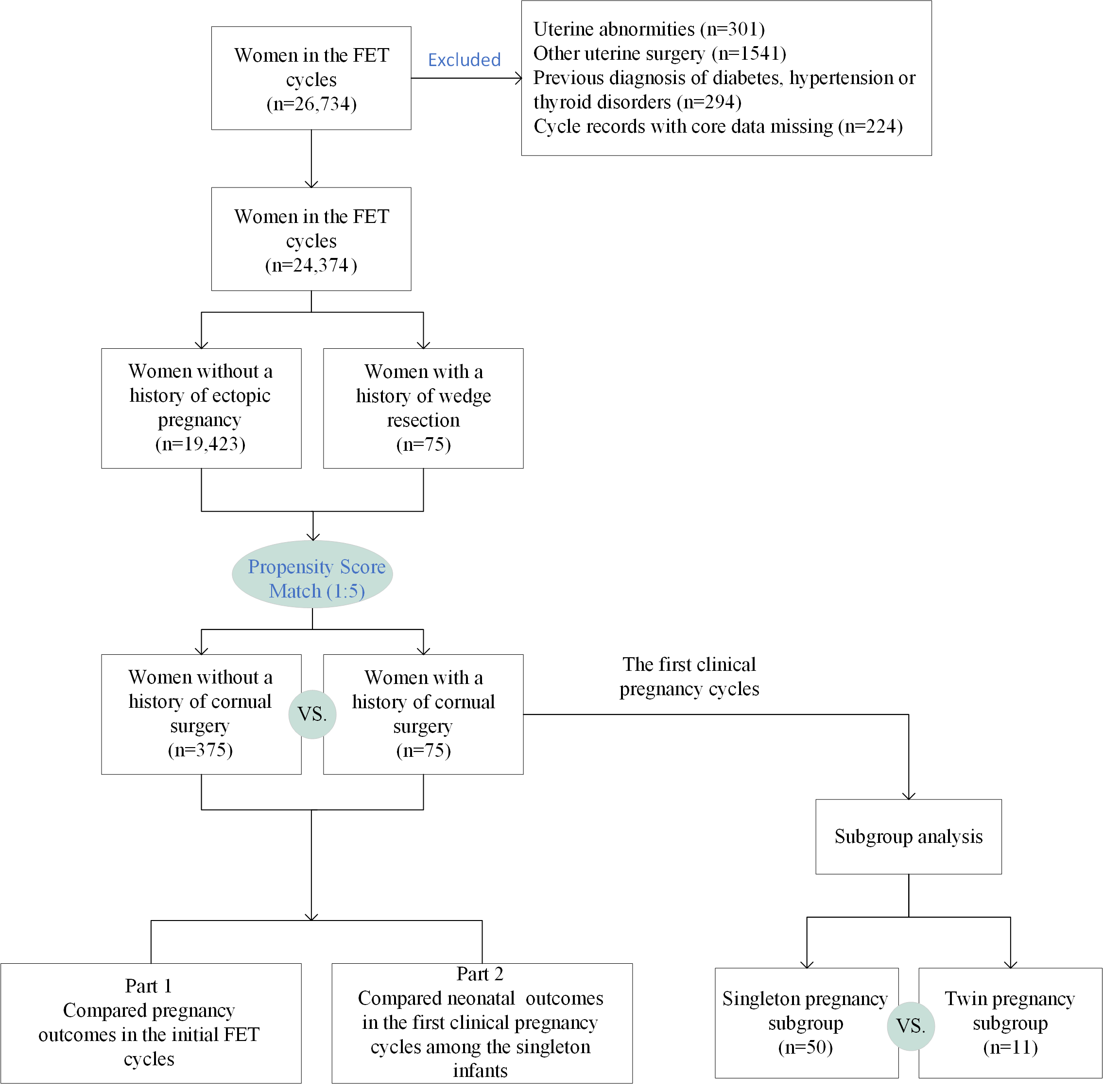

Supplement: Supplementary file 1 — Additional file 1: Supplemental Figure S1. Flow chart of the study. [file 12958_2022_896_MOESM1_ESM.tif]
